# Prevalence of diabetes mellitus among children and adolescents in the district of Abidjan in Cote d’Ivoire: a population-based study

**DOI:** 10.1186/s40200-016-0261-7

**Published:** 2016-09-20

**Authors:** Marie Laurette Agbre-Yace, Elizabeth Eberechi Oyenusi, Abiola Olufunmilayo Oduwole, Michèle Dominique Ake, Jacko Rhedoor Abodo

**Affiliations:** 1 Centre Anti-diabétique, Institut National de Santé Publique, BP V47 Abidjan, Côte d’Ivoire; 2 Paediatric Endocrinology Training Centre for West Africa, LUTH, Lagos, Nigeria; 3 Department of Paediatrics, College of Medicine, University of Lagos/Lagos University Teaching Hospital./Paediatric Endocrinology Training Centre for West Africa, LUTH, Lagos, Nigeria; 4 Pharmacie et Laboratoire de Nutrition, INSP, BP V 47 Abidjan, Côte d’Ivoire; 5 UFR Sciences Pharmaceutiques et Biologiques, Université Félix-Houphouët-Boigny, BP V 34 Abidjan, Côte d’Ivoire; 6 Service d’Endocrinologie diabétologie, CHU yopougon 23, BP 632 Abidjan 23, Côte d’Ivoire; 7 UFR Sciences Médicales d’Abidjan/Université Félix Houphouet Boigny de Cocody, Abidjan, Côte d’Ivoire

**Keywords:** Diabetes mellitus, Children, Adolescents, Abidjan, Cote d’Ivoire, Fasting blood glucose

## Abstract

**Background:**

World Health Organization has predicted a worldwide rise in the prevalence of diabetes mellitus. Cote d’Ivoire is not exempted as evidenced by such factors as obesity and sedentary life style amongst others. The objective of the study was to determine the prevalence of diabetes mellitus (DM) among children and adolescents in the district of Abidjan in Cote d’Ivoire.

**Methods:**

A cross-sectional descriptive survey using a multi-stage sampling approach was conducted from March to April 2013. 1572 children and adolescents aged 02–19 years were surveyed in 687 randomly selected households in three municipalities. Capillary fasting glucose was performed in all subjects, and when abnormal was followed by an Oral Glucose Tolerance Test (OGTT). Definitions of Impaired Fasting Glucose (IFG) and DM (Diabetes Mellitus) were according to International Society for Paediatric and Adolescent Diabetes (ISPAD) Guidelines.

**Results:**

The prevalence of DM and IFG were 0.4 % and 14.5 % respectively. There was no significant differences between patients with different glycemic status in terms of ethnicity/nationality (*p* = 0.98) or gender (0.079). In the rural areas, 565 (81.1 %) subjects were normoglycaemic and 132 (18.9 %) subjects hyperglycaemic while there were 773 (88.3 %) normoglycaemic subjects and 102 (11.7 %) hyperglycaemic subjects respectively from the urban areas of residence and this difference was statistically significant (*p* = 0.000). The prevalence of diabetes mellitus was identical (0.4 %) in the two age groups (2–9 years and 10–19 years). Seventy-seven (4.9 %) children who participated in the study had at least one diabetic parent. The proportion of participants with a diabetic father (59, 3.8. %) was twice the proportion with a diabetic mother (30,1.9 %) and this was statistically significant (*p* = 0.002). Only 10 out of 228 patients with IFG reported for the follow up OGTT and no impaired glucose tolerance was identified in these patients.

**Conclusion:**

The prevalence rate of DM among children and adolescents was 0.4 %. Nationwide awareness campaigns and prevention programmes about diabetes in childhood should be instituted and existing ones strengthened. Adequate commitment from the relevant stakeholders especially the country’s ministry of health is also advocated to stem this looming epidemic.

## Background

World Health Organization (WHO) has predicted a worldwide rise in the prevalence of diabetes mellitus (DM) that is expected to affect 300 million people by 2025 [1]. This increase is more important in developing countries particularly in sub-Saharan Africa because of adding to the burden of infectious diseases plaguing the region. Cote d’Ivoire is not exempted as evidenced by such factors as obesity and sedentary life style. An additional possible consideration is that as authorities tackle previously endemic childhood infections and malnutrition, more children are likely to survive into adulthood and may develop diabetes if the other risk factors predominate. In 2006, the International Federation of Diabetes (IDF) estimated the number of children with type 1 diabetes to be 440 000, with an annual increase of 3 % per annum and 70,000 newly diagnosed cases per year [2]. Recent data of type 2 diabetes show increase in several parts of the world also [2]. In USA, 15,000 youth are diagnosed with type 1 diabetes annually and 3700 with type 2 diabetes [3]. Individuals aged between 20 to 79 years with DM will reach approximately 812.9 thousand in Cote d’Ivoire in 2030 as predicted by IDF [2]. Previous Ivorian studies on prevalence of DM were mainly hospital based including few local surveys in schools [4]. There has been no prevalence study of DM in children and adolescents conducted in the community, hence a need to target a large number of subjects and also collect the data in a “real life” community based setting.

Therefore, this study aimed to determine the prevalence of the DM among children and adolescents in the district of Abidjan in order to provide data for possible early public health intervention.

## Methods

This was a cross-sectional study, carried out in Abidjan, the economic capital of Cote d’Ivoire, a cosmopolitan town inhabited by one fifth of the country’s population who come from different parts of the Cote d’Ivoire and West Africa. Abidjan has approximately 2,953,018 inhabitants and is divided into 13 communes and each commune is divided into neighbourhoods. The study was conducted during the period of Easter Holidays from March 29 to 04 April, 2013, by 18 teams (i.e., 2 people per team with 1 interviewer and 1 health worker) and 3 supervisors across the selected communes. The study teams had a one-day training on collection of data from children and adolescents using the survey instruments and the glucose meter for the fasting blood glucose by the principal investigator and a specialist in demography.

Ethical approval was obtained from the National Ethics and Research Committee of Cote d’Ivoire before commencement of the study.

### Sample size determination

The calculated minimum sample size was 1422. A multi-stage sampling was used to determine the population of the survey. At the first stage, three communes were chosen on the basis of the size of the highest population according to the last General Census of Population and Habitat (RGPH 1998). The communes selected are: (i) Yopougon (1039 198 inhabitants, 45 % of children 2–19 years or 467,249), (ii) Abobo (963,703 inhabitants, 46.8 % of children aged 2–19 years or 451,201) and (iii) Cocody (380,115 inhabitants, 39.4 % of children aged 2–19 years or 149,893).

At the second stage, a systematic sampling random was conducted in each commune to select 3 neighborhoods. On the basis of the list containing all the neighborhoods (INS), each of them was assigned a number. The sampling interval k was calculated by the formula N/n (the sample size is: *n* = 3 and N is the number of neighborhoods). A starting point was selected at random from the randomly selected excel numbers. The next neighborhood is 1 + k. The nine neighborhoods selected were: (i) Abobo commune: Abobo Baoule Anonkoi-Kouté, Sogefia Habitat; (ii) Cocody coomune: Sopim Valleys, Angre, Deux Plateaux II; (iii) Yopougon commune: Yopougon Kouté Sogefia Kouté Andokoi.

At the third stage, households were randomly selected. Inside of each neighborhood, one islet or zone was identified randomly. On entering the street of the islet, one house was selected by a draw and after that the subsequent household was selected using a sampling interval of 2. A directional right approach was identified starting from the randomly chosen household 1; continuing taking the 1 + k (*K* = 2) household on this direction. At the end of the street, the right turn was maintained and the interviewers continued to pick houses on the right.

If there were no residents between 2 and 19 years of age in the selected household or the selected resident did not agree to participate in the survey, the household was replaced with a household nearby. If there are no household on this direction, the interviewer targeted the household which has the nearest door to the last household interviewed. Recruited subjects were children and adolescents aged 2–19 years in whom written parental consent and accent were obtained. Sick children or children previously diagnosed with diabetes were excluded.

Two meetings were arranged, one, the day before to explain the purpose of the study and obtain the consent and, the participants were asked to remain in a fasting at least for 8 h for the next day.

### Data collection

The questionnaire was tested by a pilot survey in the community. A wide range of information including demographic characteristics, family characteristics and personal medical histories were collected. Other information collected included the diet the day before the blood glucose test. Questionnaire was also used to collect additional information for blood testing.

The fasting plasma glucose was performed for all participants with the glucose meter Accu-Chek Active according to the manufacturer’s instructions [5]. The Accu-Chek Active system meets the ISO 15197 requirements because it uses finger-stick capillary whole blood sample which is set to plasma serum standard and shows result in plasma glucose values. Capillary blood from a finger puncture was immediately analysed for fasting blood glucose (FBG) for all participants and the results filled in the questionnaire and also communicated to the participant and family on site.

Participants identified with impaired fasting glucose (IFG) were referred to the National Institute of Public Health (INSP) for further evaluation by Oral Glucose Tolerance Test (OGTT) where blood glucose was measured in the laboratory by hexokinase enzymatic method while those diagnosed with diabetes were referred for expert management.

The definition for IFG, impaired glucose tolerance (IGT) and diabetes was based on the criteria of American Diabetes Association (ADA) [6] and International Society for Pediatric and Adolescent Diabetes (ISPAD) [7]. Diabetes was diagnosed if *FPG was ≥7.0 mmol/l (126 mg/dl)* or *2 h- post glucose (h PG) was ≥ 11.1 mmol/l (200 mg/dl).* A subject was classified as having IFG if FPG is between *5.6–6.9 mmol/L* with *2hPG <7.8 mmol/*l after OGTT and IGT if 2hPG after OGTT was between 7.8–11.0 mmol/l with FPG (<5.6 mmol/l(100 mg/dl) respectively [6, 7]. The OGTT was performed as described by WHO, using anhydrous glucose of 1.75 g/kg of body weight to a maximum of 75 g dissolved in water [8].

### Data analysis

All the data were entered by using the CSPro software. Results were analyzed with SPSS 16. Data are presented as counts, frequencies and percentages. Chi-square analysis was used to compare differences between proportions. Fisher’s exact test was used to determine statistical significance when small numbers of patients were involved in analysis. Probability (*p* value) of <0.05 was taken as statistically significant.

## Results

### Household characteristics

A total of 687 households were sampled in the survey and complete data were collected on 1572 participants aged 2–19 years. Table 1 shows the distribution of the households in the selected communes. Fewer households (21.8 %) were visited in Yopougon because of availability of large numbers of eligible children. In contrast, almost double that number of households were visited in Coccody and Abobo (40.2 % and 38 % respectively). The household sizes are also shown in Table 1. Majority of the households were made up of between 5 and 10 members. In up to 13.8 % of households, more than 10 occupants lived under the same roof.

**Table 1:** Distribution of households by selected socio-demographic characteristics

Socio-demographic characteristics of household		Percentage (%)	Frequency
Commune	Yopougon	21.8	150
	Cocody	40.2	276
	Abobo	38.0	261
Area of residence	Kouté village	6.6	45
	Andokoi	6.8	47
	Sogefia-Kouté	8.4	58
	Sopim-Vallon	13.0	89
	2 Plateaux 2	14.7	101
	Angré	12.5	86
	Abobo Baoulé	12.5	86
	Anonkoi-Kouté	14.4	99
	Sogefia-Habitat	11.1	76
Household size	Less than 5 persons	25.5	175
	Between 5 and10 persons	60.7	417
	More than 10 persons	13.8	95

Socio-demographic characteristics of the parents of surveyed children and adolescents were reported by age, education, religion, ethnicity or nationality and history of diabetes as shown in Table 2. The majority of the participants’ mothers were aged between 25 and 49 years (78.7 %). In terms of the fathers, approximately 56.5 % were between 25 and 49 years. Data on the educational level of the parents showed that a small proportion (11.8 %) of them reached the level of higher education. When doing a comparison between the level of education and sex of parents, it was observed that the proportion of mothers who have reached this level of education is very low (5.1 %), while for the same level, fathers had better rates. Indeed, they represented three times (18.5 %) the frequency of mothers. In addition, it was also noted that the proportion of uneducated mothers (36.5 %) is twice that of fathers in the same situation (18.9 %).

**Table 2:** Socio-demographic characteristics of parents of children and adolescents

Demographic characteristics of parents		Father		Mother		Total	
		Percent (%)	Frequency	Percent %	Frequency	Percent %	Frequency
Age	20 - 24 years	0.1	1	4.9	34	2.5	35
	25- 29 years	2.3	16	16.0	110	9.2	126
	30 - 34 years	11.1	76	22.9	157	17.0	233
	35 - 39 years	13.7	94	19.5	134	16.6	228
	40 - 44 years	16.4	113	12.7	87	14.6	200
	45 - 49 years	13.0	89	7.6	52	10.3	141
	50 - 54 years	13.7	94	4.7	32	9.2	126
	55 - 59 years	4.9	34	1.6	11	3.3	45
	≥60 years	6.1	42	1.0	7	3.6	49
	^a^IDK (I don’t know)	18.6	128	9.2	63	1.9	191
Level of Education	None	18.9	130	36.5	251	27.7	381
	Primary	21.0	144	26.9	185	23.9	329
	Secondary	29.5	203	24.6	169	27.1	372
	tertiary	18.5	127	5.1	35	11.8	162
	No formal education	3.9	27	2.9	20	3.4	47
	^a^IDK	8.1	56	3.9	27	6.1	83
Religion	Christianity	57.5	395	61.7	424	59.6	819
	Islam	35.4	243	34.1	234	34.7	477
	Animist	1.6	11	0.1	1	0.9	12
	No religion	2.3	16	1.7	12	2.0	28
	Other religion	0.9	6	0.7	5	0.8	11
	^a^IDK	2.3	16	1.6	11	2,0	27
Ethnicity or Nationality	Akan	35.2	242	40.2	276	37.7	518
	Krou	7.6	52	7.3	50	7.4	102
	Mandé du Nord	10,2	70	10.0	69	10.1	139
	Mandé du Sud	7.9	54	8.3	57	8.1	111
	Gur	7.4	51	5.4	37	6.4	88
	ECOWAS	27.7	190	25.2	173	26.4	363
	Other African	0.7	5	1.0	7	0.9	12
	Other continent	1.0	7	0.7	5	0.9	12
	IDK	2.3	16	1.9	13	2.1	29
Diabetic history	Diabetes	3.2	22	1,9	13	2,5	35
	No diabetes	35.5	244	38,0	261	36,8	505
	IDK	61.3	421	60.1	413	60.7	834
Actual Occupation	No occupation	3.8	28	1.0	7	2.5	35
	Farmer employer	5.5	38	0.4	3	3.0	41
	Farmer employee	01	1	0.0	0	0.1	1
	Trader	11.1	76	3.,3	256	24.2	332
	Professional, Technician, Officer	23.3	160	8.4	58	15.9	218
	Service men	8.4	58	0.4	3	4.4	61
	skilled worker	2.0	14	0.4	3	1.2	17
	Unskilled worker	2.8	19	0.4	3	1.6	22
	Clerc	2.3	16	09	6	1.6	22
	Housewife	0.0	0	37.7	259	18.9	259
	Student	0.7	5	1.5	10	1.1	15
	Others	34.4	236	9.0	62	21.7	298
	IDK	3.8	36	12	17	3.9	53

^a^ *IDK* I don’t know

Table 2 also showed that the main religions practiced by the parents are Christianity and Islam with a predominance of Christian religion. The table also showed that the major ethnic groups and other nationalities living in Côte d’Ivoire were well represented. In terms of specificities, there is a dominance of the Akan people. Thus, 37.7 % of parents (35.2 % of fathers and 40.2 % of mothers) belong to this ethnic group of Côte d’Ivoire. This was followed closely by a group which includes all expatriates of the Economic Community of West African States (ECOWAS) in Côte d’Ivoire.

Approximately 2.5 % (3.8 % for fathers and 1.0 % of mothers) reported not having any occupation. In addition, other parents (0.7 % of fathers and 1.5 % of mothers) of respondents were still students. Overall, fathers were more occupied in the formal sector than mothers. Considering the occupation of the mother, the study noted that 37.3 % of mothers had a business trade compared with only 11.1 % fathers while 36.2 % of mothers are housewives. Concerning family history of diabetes, 2.5 % of parents have diabetes, 3.2 % among the fathers and 1.9 % among the mothers as shown in the table.

The age and sex distribution including the educational level of the children and adolescents surveyed are shown in Table 3. Out of the total of 1572, there were 690 (43.9 %) males and 882 (56.1 %) females giving a male: female ratio of 1:1.28. Majority of the respondents were students and those with no educational attainment were those not yet enrolled in school and those who dropped out of school as shown in the table.

**Table 3:** Socio-demographic characteristics of surveyed children and adolescents

Demographic characteristics		Male		Female		Total	
		Percent %	Frequency	Percent %	Frequency	Percent %	Frequency
Age	2 - 9 years	55.1	380	43.0	379	48.3	759
	10 -19 years	44.9	310	57.0	503	51.7	813
Level of Education	None	18.7	129	25.4	224	22.5	353
	Preschool	5.9	41	5.3	47	5.6	88
	Primary	50.3	347	43.1	380	46.2	727
	Secondary	22.8	157	23.6	208	23.2	365
	Tertiary	1.0	7	0.7	6	0.8	13
	No formal education	1.3	9	0.0	17	1.6	26

### Glycaemic profile of respondents

Table 4 shows that majority of the study subjects (85.1 %) had their blood glucose between 60 mg/dl and 100 mg /dl while 14.5 % had impaired fasting glucose. Only 6 patients had fasting blood glucose was greater than 126 mg /dl at screening giving a prevalence of approximately 0.4 % (4/1000). Further testing of patients with IFG by OGTT test was performed only in ten people (0.44 %) who kept the appointment given for the administration of the test at the National Institute of Public Health. No impaired glucose tolerance was identified in this population of children after the OGTT.

**Table 4:** Distribution of participants according to the glycemic profile (ISPAD guidelines^7^)

Glycemic profile of respondents	Male		Female		Total	
	%	Frequency	%	frequency	%	frequency
Normal fasting glycemia	83.3	575	86.5	763	85.1	1338
Impaired fasting glycemia	16.1	111	13.3	117	14.5	228
Diabetes Mellitus	0.6	4	0.2	2	0.4	6

Table 5 shows the relationship between the glycaemic profile of the study subjects and ethnicity/nationality, sex, age group, area of residence and size of households. There was no significant differences between patients with different glycemic status in terms of ethnicity/nationality (*p* = 0.98) or gender (0.079). With respect to the areas of residence, the areas were classified into urban versus rural and the glycaemic profile classified as normoglycaemic versus hyperglycaemic (IFG &DM). In the rural areas, 565 (81.1 %) subjects were normoglycaemic and 132 (18.9 %) subjects hyperglycaemic while there were 773 (88.3 %) normoglycaemic subjects and 102 (11.7 %) hyperglycaemic subjects respectively from the urban areas of residence and this difference was statistically significant(*p* = 0.000). The prevalence of diabetes mellitus was identical (0.4 %) in the two age groups (2–9 years and 10–19 years) studied as also shown in Table 6.

**Table 5:** Glycemic profile distribution by ethnicity or nationality, sex, age and area of residence

Characteristics of Respondents		Glycemic Profile			Total
		Normal fasting glycemia	Impaired fasting glycemia	Diabetes Mellitus	
Ethnicity or nationality	Akan	86.0	13.6	0.4	523
	Krou	86.6	13.4	0.0	142
	Mandé du Nord	82.7	16.8	0.5	220
	Mandé du Sud	84.7	14.5	0.8	131
	Gur	84.3	15.7	0.0	134
	CEDEAO	86.4	13.0	0.6	361
	Other African	100-0	0.0	0.0	6
	Other continent	83.3	16.7	0.0	18
	IDK	70.3	29.7	00	37
Sex	Male	83.3	16.1	0.6	690
	Female	86.5	13.3	0.2	882
Age	2 -9 years	88.0	11.6	0.4	759
	10 -19 year	82.4	17.2	0.4	813
Area of residence	Yopougon	82.9	16.9	0.2	556
	Cocody	88.8	18.1	0.2	519
	Abobo	83.3	16.3	0.4	497

**Table 6:** Relationship between glycemic profile of the children and diabetes in a parent

Glycemic Profile	Father diabetic		Mother diabetic		Total	
	Percent (%)	Frequency	Percent (%)	frequency	Percent (%)	Frequency
Normal fasting glycemia	83.1	49	80.0	24	82.0	73
Impaired fasting glycemia	16.9	10	20.0	6	18.0	16
Diabetes Mellitus	0.0	0	0.0	0	0.0	0

**Fig. 1:**
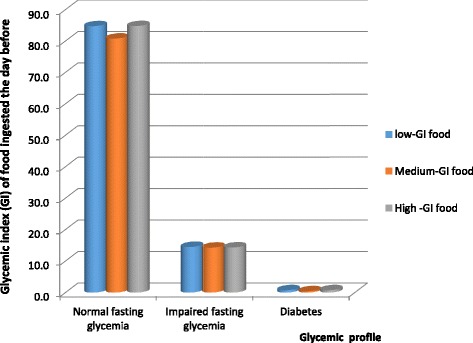
Distribution of respondents according to their glycemic status and the food consumed the day before the survey

The different groups of food according to the glycaemic index consumed by the children and adolescents the day before the test in relationship to the glycemic profile is shown in Fig. 1. It is obvious that there was no significant difference in the glycemic index of food consumed by the different participants.

The results of the study also showed that 77 (4.9 %) children who participated in the study had at least one diabetic parent. The proportion of participants with a diabetic father (59,3.8. %) was twice the proportion with a diabetic mother (30,1.9 %) and this was statistically significant (*p* = 0.002).

Table 6 showed the relationship between glycaemic profile of the subject and history of diabetes in the parents. Among the study subjects with a diabetic parent, none was diabetic but as much as 18 % of them had IFG.

## Discussion

The case prevalence of diabetes among the children and adolescents in the present study was 0.4 %, (4 per 1000). This was comparable to a Tunisian study by Ghannem et al [9] which reported a prevalence of 0.4 % amongst school children aged 13–19 years. However the observed prevalence is slightly higher than a prevalence of 0.3 % documented in a similarly designed Iranian study by Chakhadi et al. [10] among elementary school children though the age range in that study was between 6 to 12 years [10]. The prevalence was also higher than a rate of 3.2 per 1000 of diabetes among U.S. children aged less 18 years of age from data released from the National Survey of Children’s Health(NSCH) [3]. The prevalence in the US study [3] could have been under-reported because the NSCH used random-digit dialing to recruit and survey households and obtained parental response on whether the child had been diagnosed with diabetes. Similarly, the prevalence in the present study was also higher than a prevalence of 109.5 per 100,000 (1.095 per 1000) documented in a Saudi-Arabian questionnaire based study on type 1 DM by Al-Herbish et al. [11]. An added difference may be because only type 1 DM patients were reported in Saudi-Arabian study [11]. The prevalence rate in the present study was also higher than a case prevalence of 0.33/1000 in a South-Eastern Nigerian population based study by Afoke et al. [12] among school children aged 5–17 years. However an earlier report in Cote d’Ivoire by Abodo et al. [4] reported a higher prevalence rate of 2.1 % among paediatric hospital admissions. In contrast, some other hospital based prevalence rates which ranged from 0.33-2/1000 reported in studies from South-East [13], South-South [14] and North-Western [15, 16] Nigeria, respectively were lower compared to the index study. The lower prevalence rates may be related to the population and also the fact that those studies [13–16] mainly involved children with type 1 DM. Conversely, another hospital based prevalence rate of 10.1/1000 in North-Central Nigeria by John et al. [17] was noted to be higher. It is also important to note that the low turn-out of only 0.44 % of the subjects with IFG for a follow-up OGTT may have contributed to a lower prevalence of diabetes in the present study compared to the reports by Abodo et al. [4] and John et al. [17] This poor response rate may have been due to the distance between the hospital and the residences coupled with the fact that limited financial resources in the index study could not support the transport of parents and their children to the referral centre.

With regards to age and gender, there were no significant differences as has been observed in other studies [3, 9–11, 15, 17]. Even though the number of the subjects diagnosed with diabetes in the present study is small, it is however noteworthy that the male: female ratio of the diabetic subjects was 2:1 similar to a significant male preponderance of 3:1 in the study by Afoke at al and 1:0.6 by Adeleke at al respectively [12, 16]. There was no statistical difference between the different ethnic groups and nationality in the present study in contrast to the US epidemiologic study [3] which documented significant differences in that non-Hispanic white children who had a substantially higher prevalence of diabetes (3.8/1000) compared with other racial and ethnic categories (2.2/1000) [3]. Likewise the South-East Nigerian survey also reported significant differences among different locations even within the same Igbo ethnic group [12].

More participants who had deranged blood glucose lived in the rural areas of residence compared to urban areas of residence. This has also been noted by an Indian study by Rathod et al. [18] highlighting the fact that diabetes which was erstwhile associated with urban lifestyle is also becoming prevalent in the rural population. This may be because the rural masses are gradually adopting urban ways of living, feeding and sedentary lifestyles. The implication of this is that prevention campaigns and programmes should also target the rural areas of residence.

The increased proportion of diabetic fathers compared to diabetic mothers observed in the current study is similar to other reports from Ghana, Nigeria and Sierra Leone all in West Africa [19–22] and rural areas of the United Republic of Tanzania [23] which documented higher prevalence rates of diabetes mellitus in men than women in the same study areas. A United Kingdom report [24] stated that men aged 35–54 are almost twice as likely to have diabetes compared to their female counterparts. As noted in the present study, more than half of the fathers were in this age group of 35–54 years which could have also accounted for the male preponderance. A meta-analysis by Hilawe et al. [19] indicated that men who lived in the low-income countries of sub-Saharan Africa were more likely to be diagnosed with diabetes mellitus than the corresponding women. This difference between the sexes may be a consequence of differences between men and women in the distribution of risk factors for diabetes mellitus (e.g. obesity, physical inactivity, poor diet and smoking, etc.) in low-income countries [19, 25] coupled with the possibility of women in low-income countries having particularly poor access to health-care services and therefore little chance of being diagnosed with diabetes [19]. The implication of increased diabetes in men in the current study is that in many sub-Saharan African countries, men are the bread-winners and affectation by a chronic illness like DM can impact negatively on the health of the child by reducing the father’s productivity or even more seriously, the life expectancy.

The literacy level of the mothers in the present study was also noticed to be quite low compared with the fathers. This may affect the children’s health because studies have shown a strong link between the well-being of women & children and the educational level of the women [26]. Educated mothers have access to information, are socially and economically empowered thus breaking the vicious cycle of poverty, ignorance and disease.

### Limitations

The limitation of the study was the inability to perform OGTT on all the patients identified with IFG which may have affected the number of confirmed diabetic cases. Future studies are also needed in which anthropometry, insulin resistance and antibody testing can be assessed in the children diagnosed with diabetes to determine the specific type of diabetes and definitive epidemiological classification.

## Conclusion

The case prevalence of diabetes among the children and adolescents in the present study was 0.4 %. There was no significant differences between patients with different glycemic status in terms of ethnicity/nationality, gender or the two major age groups studied. Significantly more subjects were seen with hyperglycaemia from the rural areas compared to the urban areas. Impaired fasting glycaemia was seen in 14.5 % of study participants. A sizeable number of study subjects had diabetic parents with fathers being more affected than mothers. The literacy level of the parents in the study was low with mothers being affected than fathers.

Nationwide awareness campaigns about diabetes in childhood should be instituted and where already existent should be intensified so that the populace is educated appropriately regarding the signs of the disease and prevention strategies where applicable. With the IDF’s prediction of increase in incidence of diabetes in Cote d’Ivoire by 2030, adequate commitment from the relevant stakeholders especially the country’s ministry of health is advocated to stem this looming epidemic.

## References

[1] King H, Aubert RE, Herman WH (1998). Global burden of diabetes, 1995-2025: prevalence, numerical estimates, and projections. Diabetes Care.

[2] International Diabetes Federation (2011). IDF Diabetes Atlas.

[3] Lee JM, Herman WH, McPheeters ML, Gurney JG (2006). An epidemiologic profile of children with diabetes in the U.S. Diabetes Care.

[4] Abodo J, Lokrou A, Yoboué L, Sanogo A. Le diabète sucré à l’Hôpital Militaire d’Abidjan : une série ambulatoire de 473 cas. Méd Mal Métab. 2008; 2 N°6-P 639-642.

[5] *Accuchek Active®* blood glucose monitor [user’s manual] Mannheim (Germany): Roche Diagnostics; 2003.

[6] American Diabetes Association (2012). Diagnosis and classification of diabetes mellitus. Diabetes Care.

[7] Craig ME, Jefferies C, Dabelea D, Balde N, Seth A, Donaghue KC. ISPAD Clinical Practice Consensus Guidelines 2014 Compendium: Definition, epidemiology, and classification of diabetes in children and adolescents. Pediatr Diabet. 2014;15(Suppl 20):4-17. 10.1111/pedi.12186 25182305

[8] World Health Organization. Definition and diagnosis of Diabetes Mellitus and Intermediate Hyperglycemia- Report of a WHO/IDF Consultation 2006. (2005 3379 /id;World Health Organisation 1999 3377 /id) http://www.who.int/diabetes/publications/. Accessed 21st Mar 2016.

[9] Ghannem H, Harrabi I, Gaha R, Trabelsi L, Chouchene I, Essousi AS (2001). Epidemiologie du diabete chez l’enfant en milieu scolaire a Sousse, Tunisie. Diabetes Metab.

[10] Chahkandi T, Taheri F, Kazemi T, Bijari B (2015). The prevalence of diabetes and prediabetes among elementary school children in Birjand. Iran J Pediatr.

[11] Al-Herbish AS, El-Mouzan MI, Al-Salloum AA, Al-Qurachi MM, Al-Omar AA (2008). Prevalence of type 1 diabetes mellitus in Saudi Arabian children and adolescents. Saudi Med J.

[12] Afoke AO, Ejeh NM, Nwonu EN, Okafor CO, Udeh NJ, Ludvigsson J (1992). Prevalence and clinical picture of IDDM i n Nigerian Igbo school children. Diabetes Care.

[13] Ibekwe UM, Ibekwe CR. Pattern of Type 1 Diabetes Mellitus In Abakaliki, Southeastern, Nigeria. Pediatric Oncall [serial online] 2011[cited 2011 July 1]; 8. Art #48. Available From: http://www.pediatriconcall.com/Journal/Article/FullText.asp. Accessed 4th May 2016.

[14] Onyiruka AN, Phillips PO, Louis PC, Omoruyi HO (2012). Diabetes mellitus in childhood and adolescence: analysis of clinical data of patients seen in a Nigerian Teaching Hospital. Afr J Trop Med Biomed Res.

[15] Ugege O, Ibitoye PK, Jiya NM (2015). Childhood diabetes mellitus in Sokoto, North-Western Nigeria: a ten year review. Sahel Med J.

[16] Adeleke SI, Asani MO, Belonwu RO, Gwarzo GD, Farouk ZL (2010). Childhood diabetes mellitus in Kano, NorthWest Nigeria. Niger J Med.

[17] John C, Abok II, Yilgwan C (2013). Clinical profile of childhood type 1 diabetes in Jos, Nigeria. Afr J Diabetes Med.

[18] Rathod HK, Darade SS, Chitnis UB, Bhawalkar JS, Jadhav SL, Banerjee A (2014). Rural prevalence of type 2 diabetes mellitus: A cross sectional study. J Soc Health Diabetes.

[19] Hilawe EH, Yatsuya H, Kawaguchi L, Aoyama A (2013). Differences by sex in the prevalence of diabetes mellitus, impaired fasting glycaemia and impaired glucose tolerance in sub-Saharan Africa: a systematic review and meta-analysis. Bull World Health Organ.

[20] Amoah AGB, Owusu SK, Adjei S (2002). Diabetes in Ghana: a community based prevalence study in Greater Accra. Diabetes Res Clin Pract.

[21] Ejim EC, Okafor CI, Emehel A, Mbah AU, Onyia U, Egwuonwu T (2011). Prevalence of cardiovascular risk factors in the middle-aged and elderly population of a Nigerian rural community. J Trop Med.

[22] Ceesay MM, Morgan MW, Kamanda MO, Willoughby VR, Lisk DR (1997). Prevalence of diabetes in rural and urban populations in southern Sierra Leone: a preliminary survey. Trop Med Int Health.

[23] McLarty DG, Swai AB, Kitange HM, Masuki G, Mtinangi BL, Kilima PM (1989). Prevalence of diabetes and impaired glucose tolerance in rural Tanzania. Lancet.

[24] Diabetes UK Care. Connect. Campaign. Middle aged men twice as likely to have diabetes as women. https://www.diabetes.org.uk Accessed 8th July 2016.

[25] BeLue R, Okoror TA, Iwelunmor J, Taylor KD, Degboe AN, Agyemang C (2009). An overview of cardiovascular risk factor burden in sub-Saharan African countries: a socio-cultural perspective. Glob Health.

[26] Güneş PM. The Role of Maternal Education in Child Health: Evidence from a Compulsory Schooling Law. 9-24-2013 available at repository.upenn.edu/cgi/viewcontent.cgi?article = 1006&context. Accessed 10th July 2016

